# STRATEGIC-1: A multiple-lines, randomized, open-label GERCOR phase III study in patients with unresectable wild-type RAS metastatic colorectal cancer

**DOI:** 10.1186/s12885-015-1503-7

**Published:** 2015-07-04

**Authors:** Benoist Chibaudel, Franck Bonnetain, Christophe Tournigand, Marine Hug de Larauze, Armand de Gramont, Pierre Laurent-Puig, Jérôme Paget, Alexandra Hadengue, Dominique Notelet, Magdalena Benetkiewicz, Thierry André, Aimery de Gramont

**Affiliations:** 1 Division of Medical Oncology, Institut Hospitalier Franco-Britannique, 4, rue Kleber, 92300 Levallois-Perret, France; 2 GERCOR-IRC (Groupe Coopérateur Multidisciplinaire en Oncologie-Innovative Research Consortium), 151, rue du Faubourg Saint-Antoine, 75011 Paris, France; 3 Methodology and quality of life in oncology unit (EA 3181) & Quality of life and cancer clinical research platform, Hospital Saint-Jacques, 2 place Saint Jacques, 25000 Besançon, France; 4 Division of Medical Oncology, Hospital Henri-Mondor, Assistance Publique des Hôpitaux de Paris, Université Paris Est Créteil, Paris 12, 51 Avenue du Maréchal de Lattre de Tassigny, 94010 Créteil, France; 5 New drug Evaluation Laboratory, Centre of Experimental Therapeutics, Department of Oncology, Centre Hospitalier Universitaire Vaudois (CHUV), Lausanne, Switzerland; 6 INSERM U 775 - Faculté des Sciences Fondamentales et Biomédicales, Centre Universitaire des Saints-Pères, 45 Rue des Saints-Pères, 75006 Paris, France; 7 LINCOLN, 4 rue Danjou, 92517 Cedex Boulogne Billancourt, France; 8 Division of Medical Oncology, Hospital Saint-Antoine, Assistance Publique des Hôpitaux de Paris, Université Pierre et Marie Curie (UPMC), Paris VI, 184, rue du Faubourg Saint-Antoine,, 75571 Cedex 12 Paris, France

**Keywords:** Colorectal cancer, Therapeutics, Strategy, RAS, KRAS, NRAS, Clinical trial

## Abstract

**Background:**

The management of unresectable metastatic colorectal cancer (mCRC) is a comprehensive treatment strategy involving several lines of therapy, maintenance, salvage surgery, and treatment-free intervals. Besides chemotherapy (fluoropyrimidine, oxaliplatin, irinotecan), molecular-targeted agents such as anti-angiogenic agents (bevacizumab, aflibercept, regorafenib) and anti-epidermal growth factor receptor agents (cetuximab, panitumumab) have become available. Ultimately, given the increasing cost of new active compounds, new strategy trials are needed to define the optimal use and the best sequencing of these agents. Such new clinical trials require alternative endpoints that can capture the effect of several treatment lines and be measured earlier than overall survival to help shorten the duration and reduce the size and cost of trials.

**Methods/Design:**

STRATEGIC-1 is an international, open-label, randomized, multicenter phase III trial designed to determine an optimally personalized treatment sequence of the available treatment modalities in patients with unresectable RAS wild-type mCRC. Two standard treatment strategies are compared: first-line FOLFIRI-cetuximab, followed by oxaliplatin-based second-line chemotherapy with bevacizumab (Arm A) vs. first-line OPTIMOX-bevacizumab, followed by irinotecan-based second-line chemotherapy with bevacizumab, and by an anti-epidermal growth factor receptor monoclonal antibody with or without irinotecan as third-line treatment (Arm B). The primary endpoint is duration of disease control. A total of 500 patients will be randomized in a 1:1 ratio to one of the two treatment strategies.

**Discussion:**

The STRATEGIC-1 trial is designed to give global information on the therapeutic sequences in patients with unresectable RAS wild-type mCRC that in turn is likely to have a significant impact on the management of this patient population. The trial is open for inclusion since August 2013.

**Trial registration:**

STRATEGIC-1 is registered at Clinicaltrials.gov: NCT01910610, 23 July, 2013. STRATEGIC-1 is registered at EudraCT-No.: 2013-001928-19, 25 April, 2013.

**Electronic supplementary material:**

The online version of this article (doi:10.1186/s12885-015-1503-7) contains supplementary material, which is available to authorized users.

## Background

Systemic therapy is the standard practice in unresectable metastatic colorectal cancer (mCRC). Currently available chemotherapy includes fluoropyrimidine, irinotecan, and oxaliplatin either alone [1, 2] or combination [3–9]. More recently, the combination of conventional chemotherapy with molecular targeted therapies such as anti-vascular endothelial growth factor (anti-VEGF) agents bevacizumab (Avastin®) and aflibercept (Zaltrap®) or anti-epidermal growth factor receptor (anti-EGFR) agents cetuximab (Erbitux®) and panitumumab (Vectibix®) showed better treatment outcomes in the majority of mCRC patients. Moreover, data suggest that only patients with wild-type Kirsten rat sarcoma viral oncogene homolog (KRAS) and wild-type neuroblastoma RAS viral oncogene homolog (NRAS) are likely to benefit from anti-EGFR therapy [10–12].

### Irinotecan and cetuximab

FOLFIRI (regimen of irinotecan, fluorouracil, and leucovorin) in combination with cetuximab is a standard first-line regimen for patients with *KRAS* exon 2 wild-type tumors based on the results of the CRYSTAL study [13]. This combination yielded positive results in terms of response rate (RR), progression-free survival (PFS), and overall survival (OS). *KRAS* exon 2 wild-type tumors from CRYSTAL study were reanalyzed for other RAS mutations in four additional KRAS codons (exons 3 and 4) and six NRAS codons (exons 2, 3, and 4) [14]. In patients with RAS wild-type tumors, a significant benefit across all efficacy end points was associated with the addition of cetuximab to FOLFIRI. In addition, the results of the FIRE-3 study [12] confirmed previously reported results that first-line FOLFIRI-cetuximab treatment achieves higher benefit in term of OS in patients with wild-type *RAS* tumors [15]. Thus, patients whose tumors lack mutations in KRAS exons 2/3/4 and NRAS exons 2/3/4 are restricted from receiving anti-EGFR therapy. The most active approved second-line regimen in mCRC patients who failed initial irinotecan-based chemotherapy is FOLFOX-bevacizumab given until progression, based on the survival results of an Eastern Cooperative Oncology Group (ECOG) E3200 phase III trial [16]. The study was positive in terms of RR, PFS, and OS.

For patients with mCRC whose disease had progressed during second-line therapy, despite all standard therapeutic agents including anti-EGFR (for RAS wild-type tumors) and anti-VEGF, regorafenib is the only approved therapeutic option. The CORRECT study [17] showed that regorafenib provided statistically significant benefits in OS and PFS.

### Oxaliplatin and bevacizumab

Oxaliplatin-based therapy, OPTIMOX or oxaliplatin stop-and-go with fluoropyrimidines and FOLFOX-bevacizumab, is another standard first-line strategy based on the survival results of the OPTIMOX1, OPTIMOX2, and NO16966 trials [18–20]. Oxaliplatin reintroduction at first progression is a part of first-line therapy in the OPTIMOX strategy and is associated with improved survival [21]. A sensitive population with prolonged oxaliplatin-free interval is more likely to benefit from oxaliplatin reintroduction [22].

After tumor progression on full therapy (i.e., during an oxaliplatin-based sequence), second-line treatment is an irinotecan-based chemotherapy with either an anti-angiogenic agent (bevacizumab or aflibercept), as validated in the TML and VELOUR studies [23, 24], or an anti-EGFR agent (cetuximab or panitumumab) based on the results of the EPIC and ‘181’ studies [25, 26]. However these two last studies failed to show a benefit in OS. Anti-EGFR naïve patients who are resistant to standard chemotherapy can be offered cetuximab with/without irinotecan or panitumumab alone as third-line therapy [27–29]. If tumor progression occurs during third-line therapy, patients are exposed to all standard therapeutic agents, except regorafenib.

### Endpoints in strategy trials

The conduct of multiple-lines strategy trial requires the establishment of alternative endpoints that can overcome the drawbacks of OS, which remains the gold standard outcome to validate the patient clinical benefit in the framework of randomized clinical trials in oncology. Measuring OS as an endpoint in clinical trials requires a large amount of patients and long duration of follow-up to demonstrate a statistically meaningful difference between two or more treatments. Those specific requirements increase both the cost and duration of trials. In mCRC, PFS has been validated as a surrogate for OS in randomized clinical trial assessing first-line chemotherapy [30–32]. This outcome is available much earlier than OS, thus shortening the duration of trials. Moreover a smaller sample size is required to obtain PFS and demonstrate a statistical difference between two treatment arms. Although PFS was a strong surrogate for OS when assessing the efficacy of a single-line of treatment, the prediction may not be as accurate for patients receiving subsequent lines. Thus, composite endpoints such as duration of disease control (DDC) and time to failure of strategy (TFS) have been defined and evaluated to compensate for the disadvantages of the aforementioned endpoints [33, 34].

### STRATEGIC-1- Direct comparison of both strategies

STRATEGIC-1 is a randomized trial designed to determine the best sequence of therapy in patients suffering from mCRC and to define subsets of the population that will benefit most from each strategy. The study follows four successful GERCOR (Groupe Coopérateur Multidisciplinaire en Oncologie) trials evaluating the best use of available drugs: the C97-1 trial that compared FOLFIRI followed by FOLFOX and the reverse sequence, the OPTIMOX1 [6], which evaluated the concept of maintenance therapy with fluoropyrimidine alone that is oxaliplatin stop-and-go strategy [18], the OPTIMOX2 that examined the complete stop of chemotherapy [19], and the DREAM trial (OPTIMOX3) which studied maintenance therapy with targeted agents (bevacizumab +/− erlotinib) [35].

## Methods/Design

### Primary Objective

The primary objective is to demonstrate a difference in terms of DDC between the two treatment strategies: FOLFIRI-cetuximab followed by an oxaliplatin-based chemotherapy (modified FOLFOX6 [mFOLFOX6] or modified XELOX [mXELOX]) with bevacizumab vs. OPTIMOX-bevacizumab followed by an irinotecan-based chemotherapy (modified FOLFIRI3 or FOLFIRI1) with bevacizumab followed by an anti-EGFR agent (cetuximab or panitumumab) with/without irinotecan, in patients with unresectable wild-type RAS mCRC.

### Secondary Objective

The secondary objectives is to evaluate health-related quality of life (HRQoL), OS, TFS, PFS, and RR (RECIST v1.1) per sequence of therapy, DDC per drug, curative salvage surgery rate (R0 or R1 resection, global and per sequence of therapy), and safety profile of each treatment sequence.

### Trial design

STRATEGIC-1 is an international, open-label, randomized, multicenter phase III trial comparing two standard therapeutic strategies in patients with unresectable RAS wild-type mCRC. A full list of the participating institutions is displayed in Additional file 1: Table S3.

### Study schedule

The trial has started on August 2013. The estimated accrual duration is 48 months. The estimated study completion date is December 2019 (final data collection date for primary outcome measure). Survival status will be collected until the patient death.

### Coordination

GERCOR (France) is responsible for the overall coordination and management (study documents and data quality, statistical analyses). In countries other than France registration, management, and monitoring of centers are delegated to a country coordinator.

### Ethics and regulatory considerations

This study is to be conducted in accordance with globally accepted standards of the Good Clinical Practice (International Conference of Harmonization [ICH]-E6), the European Directive 2001/20/EC, the latest version of the Declaration of Helsinki, and in agreement with the Coordinated System for gaining National Health Service Permission (NIHR CSP) specific to France. The study protocol was approved for all participating centers by the French health authorities (the Agence Nationale de Sécurité du Médicament et des Produits de Santé [ANSM] on June 24, 2013 and the Independent Ethics Committee “Ile de France Paris VI” La Pitié Salpêtrière on April 12, 2013) and was registered on 25 April, 2013 at EudraCT database (EudraCT 2013-001928-19) and on 23 July, 2013 at Clinicaltrials.gov (NCT01910610). If there are any possible future substantial amendments to the original approved protocol, these have to be approved by the competent authorities. This research is part of the "Reference Methodology" (MR-001) dated January 5, 2006, relating to personal data protection. GERCOR signed a commitment to comply with the “Reference Methodology” regarding biomedical research and contracted civil liability insurance to provide patients with compensation for any injury associated with administration of the study drugs and other aspects of the conduct of the trial.

### Eligibility criteria

#### Inclusion criteria

- Signed and dated informed consent,
- Patients willing and able to comply with protocol requirements,
- Age ≥ 18 years,
- Histologically proven adenocarcinoma of the colon and/or rectum,
- Wild-type KRAS and NRAS tumor (local assessment performed either on primary tumor or metastasis). In exceptional circumstances, RAS (KRAS and NRAS) mutational status may be pending consideration at randomization provided that it is obtained within the first two cycles of first-line therapy,
- Metastatic disease according to RECIST v1.1,
- No prior therapy for metastatic disease (in case of previous adjuvant therapy, interval between the end of chemotherapy and relapse must be > 6 months for fluoropyrimidine alone or > 12 months for oxaliplatin-, bevacizumab-, or cetuximab-based therapy),
- Duly documented unresectable metastatic disease, i.e., not suitable for complete carcinological surgical resection at inclusion (patients with unresectable disease at study entry but with any potential of salvage surgery after induction therapy are eligible),
- At least one measurable or evaluable lesion as assessed by computerized tomography scan (CT-scan) or magnetic resonance imaging (MRI) according to RECIST v1.1[36],
- ECOG Performance Status (ECOG PS) between 0 and 2,
- Hematological status: neutrophils ≥ 1.5x10^9^/L; platelets ≥ 100x10^9^/L; and hemoglobin ≥ 9 g/dL,
- Adequate renal function: serum creatinine level < 150 μM,
- Adequate liver function: serum total bilirubin level ≤ 1.5x upper normal limit (UNL), serum alkaline phosphatase [ALP] level < 5xULN,
- Proteinuria < 2+ (dipstick urinalysis) or ≤ 1 g/24 h,
- Baseline evaluations performed before randomization when wild-type RAS status is known: clinical and blood evaluations no more than 14 days prior to randomization, and tumor assessment (CT-scan or MRI, evaluation of non-measurable lesions) no more than 21 days prior to randomization,
- Reliable and appropriate methods of contraception in childbearing potential women during the trial and ≤ 6 months after the end of study treatments (when applicable). Male patients with childbearing potential partner must agree to use contraception in addition to having their partner use another birth control method during the trial and until ≤ 6 months after the end of study treatments,
- Registration in France with the French National Health Care System (including couverture maladie universelle [CMU]).

#### Exclusion criteria

- Medical history or evidence of metastasis upon physical examination of central nervous system (CNS; e.g., non-irradiated CNS metastasis, seizure not controlled with standard medical therapy), unless adequately treated,
- Exclusive bone metastasis,
- Uncontrolled hypercalcemia,
- Pre-existing permanent neuropathy (National Cancer Institute (NCI) Common Terminology Criteria for Adverse Events (CTCAE) grade ≥ 2),
- Uncontrolled hypertension (defined as systolic blood pressure > 150 mmHg and/or diastolic blood pressure > 100 mmHg), or medical history of hypertensive crisis, or hypertensive encephalopathy,
- Concomitant unplanned anti-tumor therapy (e.g., chemotherapy, molecular targeted therapy, immunotherapy),
- Treatment with any investigational drug within 28 days prior to study entry,
- Other serious and uncontrolled non-malignant disease,
- Gilbert’s syndrome,
- Medical history of other concomitant or malignant disease, except for adequately treated in-situ cervical carcinoma, basal or squamous cell carcinoma of the skin, and cancer in complete remission for more than 5 years,
- Major surgical procedures (open biopsy, surgical resection, wound revision or any other major surgery involving entry into body cavity) or significant traumatic injury within the last 28 days prior to randomization, and/or minor surgical procedure including placement of a vascular device within 2 days of first study treatment,
- Pregnant or breastfeeding women,
- Patients with known allergy/hypersensitivity to any component of the study drugs,
- History of arterial thrombo and/or embolic event (e.g., myocardial infarction, stroke) within 6 months prior to randomization,
- Chronic inflammatory bowel disease,
- Total bowel obstruction,
- History of abdominal fistula, gastrointestinal perforation, intra-abdominal abscess or active gastrointestinal bleeding within 6 months prior to randomization,
- Serious, non-healing wound, active ulcer or untreated bone fracture,
- Medical history or evidence of inherited bleeding diathesis or significant coagulopathy at risk of bleeding,
- Current or recent (within 10 days of randomization) use of aspirin (> 325 mg/d), clopidogrel (> 75 mg/d), oral anticoagulants or thrombolytic agents,
- Concomitant administration of live attenuated virus vaccine such as yellow fever vaccine,
- Concomitant administration of prophylactic phenytoin,
- Treatment with sorivudine or its chemically related analogues, such as brivudine,
- Patients with known dihydropyrimidine dehydrogenase deficiency,
- Concomitant use of St John's Wort,
- Patients with interstitial pneumonitis or pulmonary fibrosis.

Each patient's eligibility will be verified by use of the standardized electronic case-report form (eCRF, LINCOLN Technologies, France).

### Interventions

**Fig. 1:**
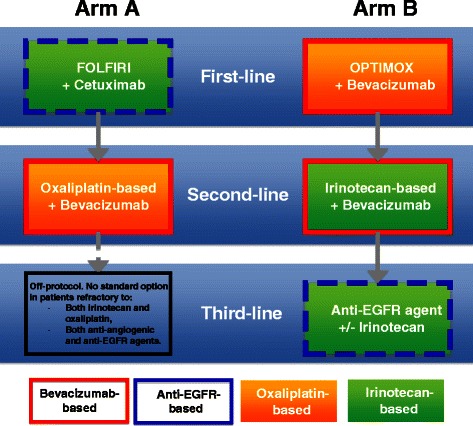
STRATEGIC-1 trial schema

Patients are exposed to all validated and recognized as standards of care agents (Fig. 1), including successive treatment lines in the mCRC therapeutic armamentarium.

A sequence of therapy starts with full therapy and may include planned/unplanned partial treatment breaks or treatment-free interval (i.e., complete stop of therapy), and ends with one of the following events (whichever occurs first): disease progression occurring on full therapy or during a partial or complete stop of therapy without any possibility of resuming full therapy. In case of progression, if the patient is not on full therapy sequence, it should be resumed in arm B (first-line oxaliplatin) if tolerable, and authorized in arm A (first-line irinotecan). Patients being under RAS mutational evaluation will not receive targeted therapy, whatever treatment arm, i.e. without cetuximab in arm A (FOLFIRI alone) and without bevacizumab in arm B (mFOLFOX7 or mXELOX alone) for a maximum of 2 cycles.

Before switching to the planned subsequent line of therapy, the following criteria must be fulfilled: at least one reason to discontinue previous line of therapy, patient general conditions compatible with treatment continuation (at investigator’s discretion), and acceptable residual toxicities from previous line of therapy.

### Arm A (Figure 1, Table 1)

**Table 1:** Treatment regimens in Arm A

A. Doses in FOLFIRI-cetuximab regimen	
H0	Cetuximab 400 mg/m^2^ /2 h IV infusion (first dose), then 250 mg/m^2^ /1 h at subsequent IV infusions, every week
	or
	Cetuximab 500 mg/m^2^ /2 h IV infusion (first dose), then 500 mg/m^2^ /1 h at subsequent IV infusions, every 2 weeks
H + 1	Irinotecan 180 mg/m^2^, in 500 ml NaCl 0.9 % solution, 1 h IV infusion
	Folinic acid 400 mg/m^2^ (leucovorin, racemic or L-form 200 mg/m^2^) in 250 ml glucose 5 % solution, 2 h IV infusion
H + 3	5FU bolus 400 mg/m^2^ in 100 ml glucose 5 % solution, 15 min IV infusion
H + 3.5	5FU continuous infusion 2400 mg/m^2^, 46 h IV infusion
B. Doses in modified FOLFOX6-bevacizumab	
H0	Bevacizumab 5 mg/kg, 30–60 min IV infusion
H + 1	Oxaliplatin 85 mg/m^2^ in 250 ml glucose 5 %, 2 h infusion
	Folinic acid 400 mg/m^2^ (racemic, or L-form 200 mg/m^2^) in 250 ml glucose 5 % solution, 2 h IV infusion
H + 3	5FU bolus 400 mg/m^2^ in 100 ml glucose 5 % solution, 15 min IV infusion
H + 3.5	5FU continuous infusion 2400 mg/m^2^, 46 h IV infusion
C. Doses in modified XELOX-bevacizumab	
H0	Bevacizumab 5 mg/kg, 30–60 min IV infusion
H + 1	Oxaliplatin 85 mg/m^2^ in 250 ml glucose 5 %, 2 h infusion
Day 1-8	Capecitabine 1250–1500 mg/m^2^ bid, from day 1 (in the evening) to day 8 (in the morning)

Cycles every 2 weeks, until disease progression, unacceptable toxicity or withdrawal of consent

### First-line

First-line treatment consists of fortnightly FOLFIRI-cetuximab until progression or unacceptable toxicity.

### Second-line

Second-line treatment consists of a fortnightly oxaliplatin-based chemotherapy (mFOLFOX6 or mXELOX) with bevacizumab until disease progression or unacceptable toxicity. The occurrence of disease progression or unacceptable toxicity during second-line therapy defines the end of treatment strategy in arm A.

### Arm B (Figure 2 and 3, Table 2)

**Fig. 2:**
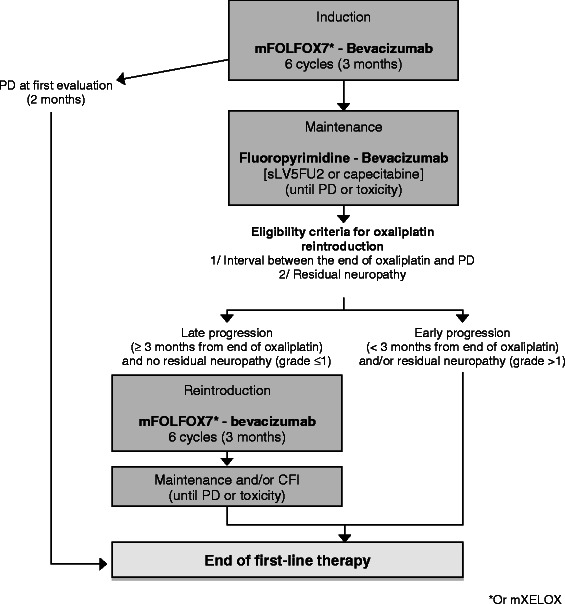
First-line OPTIMOX strategy in arm B

**Fig. 3:**
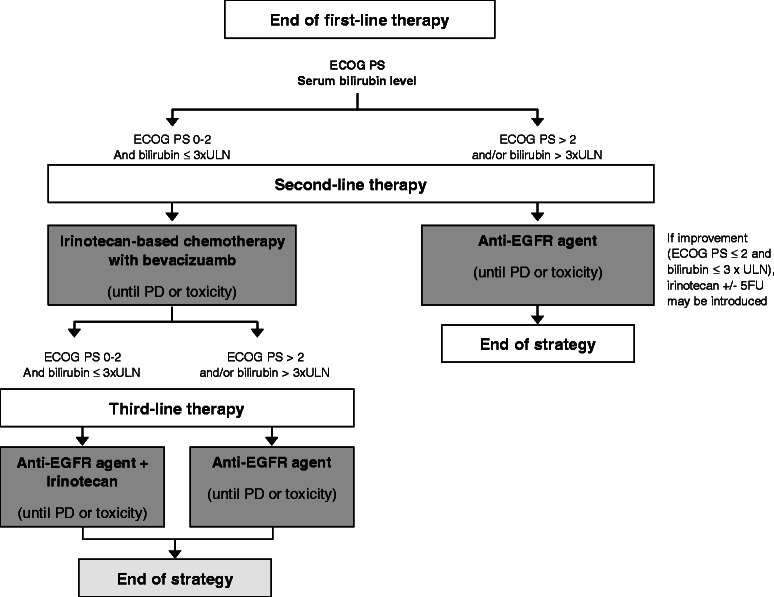
Second- and third-line strategies in arm B

**Table 2:** Treatment regimens in Arm B

A. Doses in modified FOLFOX7-bevacizumab	
H0	Bevacizumab 5 mg/kg, 30–60 min IV infusion
H + 1	Oxaliplatin 100 mg/m^2^ in 250 ml glucose 5 %, 2 h infusion
	Folinic Acid 400 mg/m^2^ (leucovorin, racemic or L*-*form 200 mg/m^2^) in 250 ml glucose 5 % solution, 2 h IV infusion
H + 3	5FU continuous infusion 3000 mg/m^2^, 46 h IV infusion
B. Doses in modified XELOX-bevacizumab regimen	
H0	Bevacizumab 5 mg/kg, 30–60 min IV infusion
H + 1	Oxaliplatin 100 mg/m^2^ in 250 ml glucose 5 %, 2 h infusion
Day 1-8	Capecitabine 1250–1500 mg/m^2^ bid, day 1 (in the evening) to day 8 (in the morning)
C. Doses in simplified LV5FU2-bevacizumab regimen	
H0	Bevacizumab 5 mg/kg, 30 min IV infusion
H + 1	Folinic acid 400 mg/m^2^ (leucovorin, racemic or L-form 200 mg/m^2^) in 250 ml glucose 5 % solution, 2 h IV infusion
H + 3	5FU bolus 400 mg/m^2^ in 100 ml glucose 5 % solution, 15 min IV infusion
H + 3.5	5FU continuous infusion 2400 mg/m^2^, 46 h IV infusion
D. Doses in capecitabine-bevacizumab regimen	
H0	Bevacizumab 7.5 mg/kg, 30 min IV infusion
Day 1-14	Capecitabine 1000 mg/m^2^ x twice daily (on days 1 to 14; 28 doses)
E. Doses in modified FOLFIRI3-bevacizumab regimen	
H0 (Day 1)	Bevacizumab 5 mg/kg, 30 min IV infusion
H + 1	Irinotecan 90 mg/m^2^ in 250 ml glucose 5 %, 1 h IV infusion
	Folinic Acid 400 mg/m^2^ (leucovorin, racemic or L*-*form 200 mg/m^2^) in 250 ml glucose 5 % solution, 2 h IV infusion
H + 3	5FU continuous infusion 2400 mg/m^2^, 46 h IV infusion
H + 46 (Day 3)	Irinotecan 90 mg/m^2^, 1 h IV infusion
F. Doses in FOLFIRI1-bevacizumab regimen	
H0	Bevacizumab 5 mg/kg, 30 min IV infusion
H + 1	Irinotecan 180 mg/m^2^ in 250 ml glucose 5 %, 1 h IV infusion
	Folinic acid 400 mg/m^2^ (leucovorin, racemic or L*-*form 200 mg/m^2^) in 250 ml glucose 5 % solution, 2 h IV infusion
H + 3	5FU bolus 400 mg/m^2^ in 100 ml glucose 5 % solution, 15 min IV infusion
H + 3.5	5FU continuous infusion 2400 mg/m^2^, 46 h IV infusion
G. Doses in cetuximab +/− irinotecan regimen	
H0	Cetuximab, 400 mg/m^2^ /2 h IV infusion (first dose), then 250 mg/m^2^ /1 h at subsequent IV infusions, every week
	or
	Cetuximab 500 mg/m^2^ /2 h IV infusion (first dose), then 500 mg/m^2^ /1 h at subsequent IV infusions, every 2 weeks
H + 1	Irinotecan 180 mg/m^2^ in 250 ml glucose 5 %, 1 h IV infusion (optional)
H. Dose of panitumumab monotherapy	
H0	Panitumumab, 6 mg/kg, 1 h IV infusion, every 2 weeks

a. b Cycles every 2 weeks, during 6 cycles (3 months). c. Cycles every 2 weeks, until disease progression, CFI, unacceptable toxicity or withdrawal of consent. d. Cycles every 3 weeks, until disease progression, CFI, unacceptable toxicity or withdrawal of consent. e. Cycles every 2 weeks, until disease progression, unacceptable toxicity or withdrawal of consent. f. Cycles every 2 weeks, until disease progression, unacceptable toxicity or withdrawal of consent. g. Cetuximab every 1 or 2 weeks, until disease progression, unacceptable toxicity or withdrawal of consent. h. Cycles every two weeks, until disease progression, unacceptable toxicity or withdrawal of consent

### First-line

First-line treatment consists of a fortnightly oxaliplatin-based chemotherapy (mFOLFOX7 or mXELOX) with bevacizumab as induction therapy during 3 months (6 cycles), followed by a maintenance therapy with fluoropyrimidine (simplified LV5FU2 regimen or capecitabine) and bevacizumab until disease progression. A chemotherapy-free interval (CFI) is allowed after at least 3 months of maintenance therapy (at investigator’s discretion) for patients fulfilling the following criteria: controlled disease (i.e., absence of tumor progression), duration of chemotherapy ≥ 6 months after starting induction treatment, baseline platelet count < 400.000/mm^3^, and normal carcinoembryonic antigen (CEA) level at first or second evaluation after starting induction therapy. In case of progression occurring 3 months or later after the last administration of oxaliplatin and in absence of residual neuropathy, maintenance therapy should be followed by reintroduction of oxaliplatin. In case of early progression (i.e., occurring less than 3 months after the last administration of oxaliplatin) and/or residual neuropathy contra-indicating oxaliplatin reintroduction, maintenance therapy should be followed by second-line therapy.

### Second-line

Second-line treatment consists of irinotecan-based chemotherapy (mFOLFIRI3 or FOLFIRI1) with bevacizumab until disease progression or unacceptable toxicity. Frail patients (ECOG PS > 2 and/or total serum bilirubin > 3xUNL) are allowed to receive an anti-EGFR agent alone (cetuximab or panitumumab).

### Third-line

At the end of second-line therapy, patients receive cetuximab with or without irinotecan or panitumumab until disease progression or unacceptable toxicity. The occurrence of disease progression during third-line therapy defines the end of treatment strategy in arm B. If the patient remains eligible for reintroduction of an oxaliplatin-based chemotherapy (i.e., no prior progression during FOLFOX or XELOX and residual neuropathy grade ≤ 2), the end of treatment strategy is defined at the date of progression after this reintroduction.

### General considerations for dose modifications

Toxicities should be graded according to the NCI CTCAE v4.0 [37]. For toxicities considered by the investigator unlikely to develop into serious or life-threatening events (e.g., alopecia, altered taste), treatment should be continued at the same dose without reduction or interruption. In addition, no dose reductions or interruptions are required for anemia (non-hemolytic) as this can be satisfactorily managed by transfusions and/or erythropoiesis-stimulating agent. If several toxicities with different grades or severities occur at the same time, dose modifications should be done according to the greatest reduction applicable. If toxicity is considered to be due solely to one of the drugs (e.g., hand-foot syndrome secondary to fluoropyrimidines, neurotoxicity due to oxaliplatin, hypertension and proteinuria due to bevacizumab, acne-like syndrome due to cetuximab or panitumumab), other drugs do not require a dose adjustment. Dosage adjustment for isolated abnormal lab values should be based on parameters at start of a treatment cycle (or one working day before). Based on the most severe toxicity experienced since the last treatment, the scheduled treatment rest period should be extended until all toxicities subside to grade 1 or less.

### Salvage surgery

Secondary surgery of metastases is authorized providing that the following conditions are respected: prior assessment of tumor response (i.e., at least one tumor evaluation after randomization) and intent to achieve a complete surgical resection (R0). In case of R0 or R1 resection, the use of a postoperative treatment and choice of the therapeutic regimen are left to the investigator’s discretion. Yet, FOLFOX is recommended in both arms. In case of R2 resection, the patient resumes the therapeutic strategy according to allocated treatment arm.

### Study endpoints

The primary endpoint is DDC, defined as the sum of PFS of each active treatment course planned in the therapeutic strategy (i.e., no disease progression at the first evaluation from the start of the sequence) [33]. DDC excludes inactive sequence (i.e., disease progression at the first tumor evaluation) and intervals between disease progression and re-initiation of treatment (either reintroduction in the stop-and-go strategy or subsequent course of treatment in the planned multi-line strategy). Censoring rules for DDC are: the end of study with no signs of progression or addition of a new (unplanned) therapeutic agent. Patients with R0 or R1 resection of metastasis are not censored for DDC.

Secondary endpoints include HRQoL, OS, TFS, PFS, and RR (RECIST v1.1) per sequence of therapy, DDC per drug, curative salvage surgery rate (R0 or R1 resection, global and per sequence of therapy), and safety profile of each treatment sequence according to the NCI CTCAE. HRQoL is assessed using the European Organization for Research and Treatment of Cancer (EORTC) Quality of Life Questionnaire (QLQ-C30). HRQoL will be considered to be improved if at least one time to HR-QoL score deterioration (five targeted dimensions) is significantly longer without a significant shorter time to HRQoL score deterioration for other four-targeted dimensions (single sufficient design). Time to HRQoL score (global health, fatigue, pain, physical and emotional functioning) deterioration will be compared between the two arms [38, 39].

Overall survival is defined as the time interval from randomization to the date of death from any cause. Alive patients will be censored at the last date known to be alive, either during study treatment period or during follow-up period. PFS is defined as the time interval from randomization to the date of first documented disease progression or death from any cause, whichever occurs first. Alive patients without progression will be censored at the last tumor assessment, either during study treatment period or during follow-up period. TFS is defined as the total PFS from the initiation of the strategy to the first of the following events: 1) death, 2) disease progression on the last received planned sequence, 3) patient requiring the addition of a new (unplanned) therapeutic agent, and 4) disease progression during a partial or complete break in therapy.

### Sample size

The following hypotheses are considered for primary analyses with two-sided type I error (alpha) of 5 %: under the null hypothesis (H_0_), DDC of the two treatment arms is equal (HR _Arm A/ Arm B_ = 1) while under the alternative hypothesis (H_1_), DDC of the two treatment arms is different (HR _Arm A/ Arm B_ ≠ 1). The sample size is planned for testing the primary variable DDC with a two-sided type I error (alpha) of 5 % and a type II error (beta) of 10 % (Software: EAST 5.3) and two planned interim analyses (to reject H0 or H1, Alpha Spending Function and O’Brien and Fleming Boundaries). A 33 % reduction in the risk of event (HR = 0.67) is assumed under the H_1_ in arm B. This reduction is estimated based on an absolute gain of 8 months for median DDC (from 16 months in arm A to 24 months in arm B) and on an assumed exponential distribution of the DDC curves. In order to observe 264 events required for the type I and II error, 450 patients will be determined for study enrolment. Besides, it is anticipated that 10 % of patients will be included without complete RAS mutational status available at randomization therefore it is foreseen that *RAS* mutation will be present in 50 % of those after randomization or will be still unknown 1 month after randomization, resulting in their exclusion. It is therefore necessary to include additional 5 % of patients. Assuming an additional 5 % dropout rate, for other reasons, 50 patients will be added in order to reach the necessary power for a statistical comparison of the DDC curves, resulting in a total of 500 patients needed to be recruited. The number of randomized patients with *RAS* mutated tumors or unknown RAS mutational status after two cycles of first-line therapy will be reviewed at the time of interim analyses. Two interim analyses will be performed after the inclusion of 150 and 385 patients without interrupting patient accrual.

In arm A, the expected median DDC is 16 months, with median PFS of 10 months for first-line treatment (FOLFIRI-cetuximab) and 6 months for second-line therapy (FOLFOX-bevacizumab). In arm B, the targeted median DDC is 24 months, with median PFS of 9 months for induction therapy, median PFS from reintroduction of 3 months (taking into account a reintroduction rate of 70 %), median second-line PFS of 7 months, and median third-line PFS of 5 months.

In regard to HRQoL, the aim is to obtain sufficient statistical power to improve median time to HRQoL score deterioration from 4 months [40] to 6 months to be considered as clinically relevant (Hazard Ratio [HR] = 0.67). A bilateral alpha type I error of 0.01 % (Bonferoni adjustment for multiple comparisons) and a power of 90 % are targeted in order to take into account the five comparisons (one for each score). To observe the 363 required events it will be necessary to have at least 1 month of follow-up among randomized patients. If only 375 patients have available score (83 %), the minimal follow-up will be 16 months.

### Randomization: sequence generation

An unblinded randomization with a 1:1 ratio is done using a minimization technique. Random allocation sequence is generated through a computer random number generator. Patients are stratified on the following parameters: center, the GERCOR prognostic score based on ECOG PS and serum lactate dehydrogenase (LDH) level (low vs. intermediate vs. high risk group) [41], prior use of oxaliplatin in adjuvant setting (yes vs. no), and extension of metastatic disease (liver only vs. other).

The minimization algorithm takes into account already randomized patients in order to allocate a new treatment. A subgroup of patients who present the same stratification variables that the patient to be randomized is isolated. The total number of patients in that subgroup is counted by stratification variables and treatment group. A less represented treatment group is selected by the system and attributed to the patient. The randomization result provided by the system is attributed in 80 % of the cases; otherwise the other treatment is attributed.

### Implementation of randomization

All investigators and on-site clinical research associates are provided with unique user names and passwords in order to access, review, and approve the eCRFs. Once randomization is started, the allocated arm appears on the randomization form and a confirmation email is sent to all members of the investigational site and of GERCOR. From then on, the randomization form is frozen and can no longer be modified.

### Statistical analysis

The statistical analysis plans (final and dedicated to HRQoL analyses) will be agreed and written before the database is frozen.

The modified (m) intent-to-treat (ITT) 1 population for efficacy analyses includes all randomized patients with locally confirmed wild-type RAS status, according to the treatment group allocated by randomization. Patients for whom RAS mutational status (wild-type or mutated) is available after 2 cycles will not be included in the analyses. This population is the primary population for all efficacy parameters (except HRQoL).

The mITT2 population that includes patients belonging to the mITT1 population with at least one QLQ-C30 completed at baseline will be used for HRQoL analyses. The safety population (all patients who received at least one dose of any planned study treatment) will be used for reporting the safety and treatment exposure data. Selected efficacy analyses will be repeated in the per-protocol (PP) population (i.e., subset of the mITT1 population meeting the following criteria: all eligibility criteria fulfilled, at least one dose of allocated treatment administered, and RAS wild-type tumor confirmed after central assessment).

All tests will be performed at a two-sided 5 % significance level with the exception of tests for the primary endpoint for which a Lan-DeMets alpha spending function with O’Brien and Fleming boundaries (function depending on the information fraction in the ITT population) will be used. The nominal significance levels for the interim and final analyses of DDC will be derived from the later function.

All tests in HRQoL analyses will be performed at a two-sided 1 % significance level. If confidence intervals (CIs) are to be calculated, those will be at a two-sided 95 % CI and a nominal (1-alpha) 100 % CI for all primary endpoints.

### Continuous Variables

Continuous variables will be summarized using descriptive statistics, i.e. number of patients with available data (n), mean, median, standard deviation (SD), 25 %-75 % quartile (Q1-Q3), minimum, and maximum. Continuous variables could be transformed to categorical variables using the median or using conventional cut-offs from bibliography or clinical practice.

### Categorical Variables

Frequencies in tables will be presented by arm, total frequency, percentages, and missing modality. Qualitative variables will be summarized by means of counts and percentages. Unless otherwise stated, the calculation of proportions will be based on the sample size of the population of interest.

### Time to Event Variables

Kaplan Meier curves will be used to describe event-free rates over time. Median event-free times by treatment arm will be reported with 95 % CIs, if the number of events allows the estimation of the median. Confidence interval of median survival time will be calculated according to Brookmeyer and Crowley[42]. Event rates at specified time points will be estimated from the Kaplan-Meier curve. The standard error will be estimated by the Greenwood formula and the log-log transformation will be used to compute CIs. The treatment effects will be summarized by means of a HR derived from a Cox proportional hazard model with its associated 95 % CI.

### Follow-up

Follow-up will be estimated using the reverse Kaplan-Meier method, and will be described using the median with its 95 % CI.

### Survival

Survival will be estimated using the Kaplan-Meier method, and will be described using the median with its 95 % CI. Univariate Cox proportional hazard model will be used to estimate HR (control arm vs. investigational arm) with 95 % CI. Multivariate Cox analysis will be done. A univariate selection procedure will serve to identify eligible explanatory variables with univariate Cox (using Wald Test) p-value lower than 0.10 as potential prognostic value.

### Pre-specified subgroup analysis

The goal of the pre-specified subgroups analyses is to confirm consistency of the impact of the arm B strategy on DDC.

### Methodology

Pre-specified subgroups are defined as follows:

- Stratification factors: center, the GERCOR prognostic score, prior use of oxaliplatin in adjuvant setting, extension of metastatic disease (liver only vs. other),
- Patient characteristics:
  - Demographic: age (< 65 vs. ≥ 65), sex (male vs. female), countries (in case of multinational participation),
  - Baseline characteristics: ECOG PS (0 vs. 1 vs. 2), prior hypertension, number of metastatic sites (1 vs. > 1), disease confined to liver (yes vs. no), location of primary tumor (colon vs. rectum vs. both), synchronous vs. metachronous disease, prior adjuvant chemotherapy (yes vs. no), LDH level (normal vs. > 1xULN), ALP level (normal vs. > 1-3xULN vs. > 3-5xULN), serum CEA level (normal vs. > 1-10xULN vs. > 10-100xULN vs. > 100xULN), white blood cell [WBC] count (< 10,000/mm^3^ vs. ≥ 10,000/mm^3^), and platelets (< 400,000/mm^3^ vs. ≥ 400,000/mm^3^),
  - Demographic: age (< 65 vs. ≥ 65), sex (male vs. female), countries (in case of multinational participation),
  - Baseline characteristics: ECOG PS (0 vs. 1 vs. 2), prior hypertension, number of metastatic sites (1 vs. > 1), disease confined to liver (yes vs. no), location of primary tumor (colon vs. rectum vs. both), synchronous vs. metachronous disease, prior adjuvant chemotherapy (yes vs. no), LDH level (normal vs. > 1xULN), ALP level (normal vs. > 1-3xULN vs. > 3-5xULN), serum CEA level (normal vs. > 1-10xULN vs. > 10-100xULN vs. > 100xULN), white blood cell [WBC] count (< 10,000/mm^3^ vs. ≥ 10,000/mm^3^), and platelets (< 400,000/mm^3^ vs. ≥ 400,000/mm^3^),
- Reintroduction rate of FOLFOX-bevacizumab in arm B (global and per center: < 40 % vs. 40 %-50 % vs. 50 %-60 % vs. ≥ 60 %).

### Analyses

Association between pre-specified subgroups and survival will be explored using univariate Cox analysis for all parameters. The continuous variables will be treated as quantitative and qualitative data using cut-off in anticipation of elaborating a practical clinical tool. Proportional hazard assumption will be graphically assessed.

Factors will be considered for inclusion in the model as potentially associated with DDC and OS if the univariate p-value is ≤ 0.1.

The Cox regression model will be used for multivariate analysis of prognostic factors for DDC and OS.

Interactions between treatment and each subgroup will be tested at a two-sided 10 % level (i.e., a p-value > 0.1 indicates no evidence of heterogeneity of treatment effect across the subgroups for each factor).

Within each subgroup, the treatment effect HR and its (1-α) % CI will be estimated using a Cox proportional hazard model on patients of this subgroup.

### Translational research

Tumor tissue and peripheral blood samples will be collected at several time points for translational research. Those samples will be used to discover and validate prognostic and predictive markers of response to anti-EGFR agents and to evaluate relationship between key angiogenic markers and clinical outcome parameters.

Prospectively collected biological material from either primitive tumor or metastatic disease will be stored until the end of patients’ accrual. All tumors will be characterized by the most frequent tumor genes alterations (including KRAS, BRAF, NRAS, PIK3CA, APC, SMAD4, FBXW7, and any prognostic and predictive relevant genes) using deep sequencing at the end of enrolment.

Whole blood samples will be drawn for subsequent extraction of DNA and RNA from lymphocytes and plasma. Samples will be collected in both arms before cycles 1 and 2 of first-line treatment and in arm B before cycles 1 and 2 of third-line treatment. A systematic translational projects with 1) validation of the micro RNA hsa-mir-31-3p as a marker of efficacy of cetuximab [43], 2) estimation of the prognostic and the predictive role of circulating tumor DNA at the time of inclusion, and 3) evaluation of differences in tumor circulating DNA between cycles 1 and 2 of treatment will be performed. DNA extracted from tumors will be used to characterize the most frequent mutations.

## Discussion

The increasing number of new therapeutic options provides more complicated treatments algorithms in patients with unresectable mCRC. Therefore, randomized strategy trials are indispensable to compare many possible treatment options in clinical practice and to make the best strategy choice that can be formally recommend in this setting. Although several randomized first-line trials (FIRE-3[12], PEAK[44], CALGB8503[45]) were designed to evaluate chemotherapy with anti-VEGF and anti-EGFR agents, the subsequent lines of treatment in these studies were not fixed and crossover was likely to pose an obstacle to prediction of OS.

## Conclusion

STRATEGIC-1 phase III study has been designed to seek for the optimal treatment sequence to be used as standard practice strategy for RAS wild-type mCRC. The trial is based on the concept of a central symmetry that is comparing two planned therapeutic multiple-line strategies each including all the currently available chemotherapy and molecular targeted agents, but in a different order. Besides, the trial aims to identify patient population that would benefit the most from anti-EGFR and anti-VEGF therapy. The study has started in July 2013 in France. The trial will be available to participants in other countries in the near future.

## Additional file

Additional file 1: Table S3. A list of the participating institutions.

## Abbreviations

VEGF: Vascular Endothelial Growth Factor
EGFR: Epidermal Growth Factor Receptor
CFI: Chemotherapy-Free Interval
CRC: Colorectal Cancer
mCRC: Metastatic Colorectal Cancer
DDC: Duration of Disease Control
TFS: Time to Failure of Strategy
PFS: Progression-Free Survival
OS: Overall Survival
RR: Response Rate
ECOG PS: Eastern Cooperative Oncology Group Performance Status
eCRF: Electronic Case Report Form
HR: Hazard Ratio
CI: Confidence Interval
RECIST: Response Evaluation Criteria in Solid Tumors
ICH: International Conference of Harmonization
ITT: Intention-To-Treat
KRAS: Kirsten Rat Sarcoma viral oncogene homolog
NRAS: Neuroblastoma RAS viral oncogene homolog
NCI CTCAE: National Cancer Institute Common Terminology Criteria for Adverse Events
PD: Progressive Disease
CNS: Central Nervous System
PP: Per-Protocol
CT-scan: Computerized Tomography-scan
MRI: Magnetic Resonance Imaging
ALP: Alkaline Phosphatase
CEA: Carcinoembryonic Antigen
LDH: Lactate Dehydrogenase
HRQoL: Health-Related Quality of Life
QLQ-C30: Quality of Life Questionnaire-C30
UNL: Upper Normal Limit
5FU: 5-Fluorouracil
FOLFIRI: Folinic acid, 5FU and irinotecan
FOLFOX: 5FU, leucovorine and oxaliplatin
LV5FU2: Folinic acid and 5FU
sLV5FU2: simplified LV5FU2
XELOX: Capecitabine and oxaliplatin
mXELOX: modified XELOX
