# Supplementary material for: STRATEGIC-1: A multiple-lines, randomized, open-label GERCOR phase III study in patients with unresectable wild-type RAS metastatic colorectal cancer
Source: BMC Cancer. 2015 Jul 4;15:496. doi: 10.1186/s12885-015-1503-7 (PMC4490616; doi:10.1186/s12885-015-1503-7)
Supplement: Additional file 1: Table S3. — A list of the participating institutions. [file 12885_2015_1503_MOESM1_ESM.docx]

**Table 3.** A list of the participating institutions.

| List of the participating institutions |
| --- |
| Hôpital Foch  Oncologie médicale  40, rue Worth  92151 SURESNES |
| CHITS – Hôpital Sainte Musse  Service d’oncologie et hématologie  54, rue Henri Sainte Claire Deville  83100 TOULON |
| Hôpital Périgueux  Service d’hépato-gastroentérologie  80, avenue G. Pompidou  24019 PERIGUEUX |
| Centre Hospitalier de Sens  Service d’oncologie  1, avenue Pierre de Coubertin  89100 SENS |
| Institut Hospitalier Franco-Britannique  4, rue Kléber  92300 LEVALLOIS PERRET |
| Hôpital Tenon - Service oncologie  4, rue de la Chine  75970 PARIS Cedex 20 |
| Hôpital Louis Pasteur  4, rue Claude Bernard  28630 Le COUDREY |
| CHD Vendée  Boulevard Stéphane Moreau  85925 La Roche sur Yon Cedex 9 |
| Hôpital Beaujon  100 Bd du Général Leclerc  92118 CLICHY CEDEX |
| Service d’Hépato-Gastroentérologie  Hôpital Saint Louis  1 avenue Claude Vellefaux  75010 PARIS |
| Hôpital Privé Jean Mermoz  Institut de Cancérologie  55 Av Jean Mermoz  69373 LYON Cedex08 |
| Clinique Armoricaine de Radiologie  21 rue du Vieux Séminaire  22015 Saint-Brieuc Cedex |
| Hospices Civils de Colmar  39 av de La Liberté  68024 COLMAR |
| Centre Hospitalier Layné  Avenue Pierre de Coubertin  40024 MONT DE MARSAN cedex |
| Hôpital Pitié-Salpêtrière  Service d’hépato-Gastroentérologie  47-83 Boulevard de l’Hôpital  75651 Paris Cedex 13 |
| Hôpital Henri Mondor  51 Avenue du Maréchal de Lattre de Tassigny  94010 Créteil |
| Centre Ste Catherine de Sienne  Oncologie médicale  2 rue Eric Tabarly BP 20215 44202 NANTES CEDEX 02 |
| Hôpital St Joseph  185 rue Raymond Losserand  75674 PARIS Cedex 14 |
| Hôpital Nord  Chemin des Bourrely  13915 Marseille Cedex 20 |
| Service d’oncologie médicale  Centre Georges François Leclerc  1 rue du Professeur Marion  21000 DIJON |
| Clinique Générale  15 rue Jacques Delpeuch  26000 VALENCE |
| Centre d'oncologie de Gentilly  2 rue Marie Marvingt  54000 Nancy |
| Institut Mutualiste Montsouris  Département d’Oncologie Médicale  42 Bd Jourdan  75014 Paris |
| Centre hospitalier de DAX, Hôpital La Côte d'Argent  Bd Yves Dumanoir  40107 DAX |
| Centre hospitalier de Cannes  Service de médecine interne oncologie  15 av des brousailles CS500008  06400 Cannes |
| Service de Gastroentérologie  Hôpitaux de Léman  3, avenue Dame  74200 THONON LES BAINS |
| Hôpital Privé de Villeneuve d’Asq  Institut de Cancérologie  20 avenu de la reconnaissance  59657 VILLENEUVE d’ASQ |
| Groupe Hospitalier Public du Sud de l’Oise (GHPSO)  Site Senlis  Rue Paul Rougé  60300 Senlis  & Site de Creil  Boulevard Laennec  60109 Creil Cedex |
| Hôpital Du Scorff  5, Avenue Choiseul  56100 LORIENT |
| STRASBOURG ONCOLOGIE LIBERALE  184 Route de la Wantzeneau  67000 Strasbourg |
| Centre Hospitalier d’Auxerre  Service d’oncologie médicale  2, boulevard de Verdun  89011 AUXERRE Cedex |
| Hôpital Européen  6, rue Désirée Clary  13331 Marseille cedex 03 |
| Centre Hospitalier  Service d’oncologie médicale  216, avenue de Verdun  36000 CHATEAUROUX |
| Hôpital Privé de l’Estuaire  Oncologie médicale  505 rue Irène Joliot Curie  76620 LE HAVRE |
| Institut d’Oncologie Hartmann  38, rue de Villiers  92300 LEVALLOIS PERRET |
| Clinique de l’Alliance  1, boulevard Alfred Nobel  37540 Saint Cyr / Loire |
| Institut de Cancérologie Lucien Neuwirth  108bis, avenue Albert Raimond  42271 Saint Priest en Jarez cedex |
| Centre d’oncologie et de radiothérapie du Parc  18, avenue du Général de Gaulle  21000 DIJON |
| Hôpital Broussais – CH Saint Malo  Hépato-gastroentérologie cancérologie digestive  1, rue de la Marne  35400 SAINT MALO |
| Centre Hospitalier Annecy Genevois (site d’Annecy)  1, avenue de l’hôpital Metz – Tessy – BP 90074  74374 PRINGY CEDEX |
| Clinique Victor Hugo  Service d’oncologie - radiothérapie  18, rue Victor Hugo  72000 LE MANS |
